# Personals

**Published:** 1913-10

**Authors:** 


### PERSONALS.

*Announcements of removal, travel, and other matters of interest are requested. Please report errors in the listing of any physician in the State and other directories that we may co-operate with the State Society in securing a correct list.*

Dr. Albert Bowen has returned to Rochester to practice, being at 221 Oxford street.

Dr. W. Eugene Powell has opened an office at 251 Arnett Boulevard, Rochester, N. Y.

Dr. W. B. Jones of Rochester has returned from an extended trip abroad.

Dr. Wm. G. Taylor of Buffalo has returned from Europe.

Dr. Charles W. Hennington of Rochester spent August at Point Abino, Canada.

Dr. Walter L. Mattick, formerly House Physician at Bellevue Hospital, announces the opening of an office at 290 Highland avenue, Buffalo, with equipment for doing general laboratory work for the profession.

Dr. Lucy A. Kenner has been appointed corporation physician to the Larkin co. of Buffalo, this firm being one of a very few in the entire country employing a physician to devote the entire time to the corporation's employees.

Dr. Albert J. Colton of Buffalo has moved to 27 Jewett avenue.

Dr. F. G. Moehlau of Buffalo has moved to 237 E. Utica St.

Drs. Roswell Park and Roland Meisenbach of Buffalo gave a dinner at the Country Club August 27, in honor of the medical delegates to the International Congress of School Hygiene. About thirty-five were present.

Dr. Harry Mead of Buffalo returned from Europe August 26.

Dr. Regina Flood Keyes of Buffalo has moved her office and residence, to 432 Delaware avenue.

Dr. N. Kavinoky and his wife, Dr. Nadina R. Kavinoky, returned September 1st from a five months' trip to Europe, devoted especially to surgical study in Berlin.

---

Dr. Geo. C. Sincerbeaux of Auburn spent a vacation of two weeks among the Thousand Islands.

---

Dr. S. E. Austin of Auburn enjoyed a two weeks' fishing trip in the Adirondacks.

---

Dr. John H. Grant, formerly of Buffalo, has moved from 202 W. 79th street to 936 West End avenue, New York City.

---

Dr. S. A. Dunham of Buffalo spent a couple of weeks on his farm near Warren, Pa., about the first of September.

---

The editor acknowledges the hospitality of Dr. A. E. Bartoo of Wilson, formerly of Buffalo.

---

Dr. John Ragone of Buffalo spent three weeks of September in northern Canada.

---

Dr. Marshall Clinton of Buffalo returned early in September from a trip to Connecticut.

---

Dr. Charles E. Flagg of Buffalo traveled through the St. Lawrence, Muskoka and Georgia Bay country in August.

---

Dr. L. H. Willis of Rochester has letters showing him to be the original of "Laurie" in Little Women.

---

Dr. A. C. Callahan of Buffalo was chairman of the parade committee of the Moose, during their Harvest Carnival in September.

---

Dr. C. F. Chaffe of Rochester has moved to 326 Park avenue.

---

Dr. R. M. Root of Buffalo has returned from Europe.

---

Dr. F. E. Fronczak, Health Commissioner of Buffalo, attended the meeting of Polish Physicians in Detroit, September 18-20

---

Dr. Isaac Sernoffsky of Buffalo has gone to Europe.

